# The ‘dnet’ approach promotes emerging research on cancer patient survival

**DOI:** 10.1186/s13073-014-0064-8

**Published:** 2014-08-26

**Authors:** Hai Fang, Julian Gough

**Affiliations:** Computational Genomics Group, Department of Computer Science, University of Bristol, The Merchant Venturers Building, Bristol, BS8 1UB UK

## Abstract

We present the ‘dnet’ package and apply it to the ‘TCGA’ mutation and clinical data of >3,000 patients. We uncover the existence of an underlying gene network that at least partially controls cancer ‘survivalness’, with mutations that are significantly correlated with patient survival, yet independent of tumour origin and type. The survivalness network has natural community structure corresponding to tumour hallmarks, and contains genes that are potentially druggable in the clinic. This network has evolutionary roots in Deuterostomia identifying *PTK2* and *VAV1* as under-valued relative to more studied genes from that era. The ‘dnet’ R package is available at http://cran.r-project.org/package=dnet.

**Electronic supplementary material:**

The online version of this article (doi:10.1186/s13073-014-0064-8) contains supplementary material, which is available to authorized users.

## Background

Cancer is a heterogeneous disease that differs phenotypically between tissues and cells of origin. Cancer is also a genomic disease in which genetic/epigenetic mutations contribute to tumour progression and heterogeneity. This heterogeneous nature poses a great challenge for cancer research, but consensus has been reached for a handful of ‘hallmarks’ [1], mostly in the underlying biology. There is a pressing need to seek consensus on more clinical aspects for the benefit of patient healthcare [2]. In this aspect, cancer genomic mutation data can be useful, especially when analysed in combination with clinical data on patients [3,4]. One of the key hopes is to provide better-informed prognostics by understanding the molecular basis of cancer patient ‘survivalness’.

Conventional research into survivalness has focused on individual tumour types, wherein cancer patients of the same type are first stratified into subtypes according to genomic data (mostly expression data [5–9], and now mutation data [10]), and then are correlated with survival data in the hope of discovering clinically meaningful subtypes. Some are successful but many are not. This data-driven unsupervised strategy is popular partly due to a lack of clinical data in parallel with genomic data. On the other hand, an increasing availability of both mutation and clinical data for multiple tumour types requires hypothesis-driven approaches to examine cancer patient survivalness. This can be seen in projects such as ‘The Cancer Genome Atlas’ (TCGA) [11], which provides multi-cancer survival data in addition to genomic mutational data.

We propose that patient survivalness can also be addressed at cross-tumour levels. Like the cancer hallmarks, there might exist common molecular programs (likely acting as gene networks) controlling cancer patient survivalness, irrespective of tumour type, age and gender. Patient survivalness is probably not merely a statistical product of correlation with survival time, but also is a cumulative outcome of mutated genes that are rooted in their evolutionary history. Any attempts to address these questions will reshape our clinical practice in cancer prognosis, diagnostics and even therapy.

To promote hypothesis-driven research on survivalness, we present an integrative approach called ‘dnet’. This method supervises both mutation and survival data, in the context of prior knowledge of the network, to search for a core gene network controlling cross-tumour cancer patient survival. This survival network is robust to data removal and is statistically significant as estimated under data randomisation. On evaluating performance on survival gene identifications, our method is superior to existing network-based methods (although most of them are not designed for this purpose). Via integrating with ontology and evolution knowledge, *dnet* is also able to clarify the survival network with relevance to: prognostic and druggable power, tumour heterogeneity and commonality, and evolutionary origins. The ‘dnet’ approach thus represents a significant advance in emerging research on cancer patient survivalness, especially in an ever-maturing era of personalised medical genomics.

## Methods

**Figure 1:**
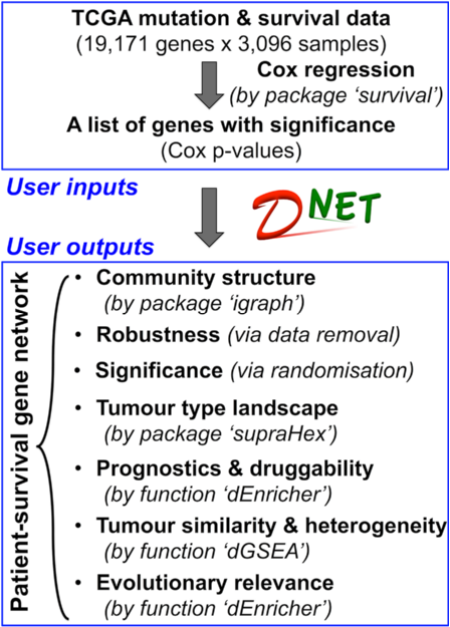
**Schematic workflow of dnet in discovering and interpreting the patient-survival gene network.**

All analytical methods used in this study have been implemented in the R package ‘dnet’ [12], for which some of functionalities depend on the other two R packages ‘igraph’ [13] and ‘supraHex’ [14]. The *dnet* package is an open-source R package that is specifically designed to analyse high-throughput biological data in an integrative manner. It has the focus on making sense of these digitised data from different angles including: integration with molecular networks, enrichments using ontologies, and relevance to gene evolutionary ages. As a proof of principle, we apply the *dnet* approach for discovery and interpretation of a core gene network controlling the patient survival across human cancers. Without loss of generality, we use this application to illuminate the *dnet* approach. Figure 1 provides the workflow of the *dnet* in discovering and interpreting the cancer-patient gene network, and is detailed below. In the package website, a step-to-step demo [15] is also provided which allows the user to completely reproduce the work in this paper.

### Survival analysis of TCGA mutation and survival data in patients of different tumour types

TCGA mutation and survival data were obtained from the supplemental tables published in [16], retaining for analysis only cancer patients with survival information available. This results in somatic mutational profiles for 3,096 patients with mutations in 19,171 genes (mapped to the Entrez Genes). These patient samples belong to one of 12 major tumour types, including bladder urothelial carcinoma (BLCA, n = 92), breast adenocarcinoma (BRCA, n = 763), colon and rectal carcinoma (COAD/READ, n = 193), glioblastoma multiforme (GBM, n = 275), head and neck squamous cell carcinoma (HNSC, n = 300), kidney renal clear cell carcinoma (KIRC, n = 417), acute myeloid leukaemia (LAML, n = 185), lung adenocarcinoma (LUAD, n = 155), lung squamous cell carcinoma (LUSC, n = 171), ovarian serous carcinoma (OV, n = 315) and uterine corpus endometrial carcinoma (UCEC, n = 230). For each patient, somatic mutations are represented as a profile of genes, in which non-zero entry indicates the number of mutations occurring in a gene. To better manage associated information on samples and genes, this dataset is provided as an instance of ‘ExpressionSet’ R object, and is available as package built-in datasets [17]. From this data representation, both clinical survival sample information and detailed gene information are readily accessible. Another advantage of doing this is to help the script coding for the survival analysis (see the demo [15]).

Survival analysis was applied [18] to TCGA mutation and survival data based on the Cox proportional hazards model. Cox regression yields an equation for the hazard as a function of explanatory variables, here including: a mutational variable for a gene in subject (a gene mutation vector containing mutation numbers across patients), and three clinical covariates (age, gender and tumour type). The baseline regression only considers three clinical covariates, and the full regression fits all these four explanatory variables. Likelihood ratio tests (LRT; analogous to the sequential ANOVA) are used to compare the full regression against the baseline regression, for the calculation of Cox hazard ratio (HR) and associated *P* value for each gene. The calculated Cox HR and *P* value are indicative of prognostic value: the extent to which mutation status for a gene correlates with patient survival advantage (after adjusting for age, gender and tumour type).

### Identification of the patient-survival gene network

The *dnet* package takes as input a list of genes with the significance information (here Cox *P* values calculated by survival analysis above), and superposes these genes onto a gene interaction network [19]. From this network with imposed node information, *dnet* sets up the pipeline to search for a maximum-scoring subgraph (the patient-survival gene network reported in this study). Given the threshold of tolerable *P* value, it gives positive scores for nodes with *P* values below the threshold (nodes of interest), and negative scores for nodes with threshold-above *P* values (intolerable). After score transformation, the search for a maximum scoring subgraph is deduced to find the connected subgraph that is enriched with positive-score nodes, allowing for a few negative-score nodes as linkers. This objective is met through minimum spanning tree finding and postprocessing, previously used as a heuristic solver of prize-collecting Steiner tree problem [20]. The solver is deterministic, only determined by the given tolerable *P* value threshold. For identification of the subgraph with a desired number of nodes, an iterative procedure is also developed to fine-tune tolerable thresholds. This explicit control over the node size may be necessary for guiding follow-up experiments. For details on the implementation, the reader is referred to the Reference Manual [12].

For the understanding of network structure, communities in the network are detected via a spin-glass model and simulated annealing (implemented in the ‘igraph’ package). Significance (*P* value) of communities is evaluated by two-sample Wilcoxon tests on two types of node degrees: within-community degrees *versus* between-community degrees.

### Robustness of the patient-survival gene network under data removal

The robustness of the patient-survival gene network (or called ‘the target network’) is assessed by removal of one tumour type per run and re-identification of networks (called ‘networks under removal’). This systematic removal is to test the robustness against the algorithm used as well as the input data (especially the potential dominance by one tumour type). To ensure the fairness of the assessment, each network, under removal, has the same/approximate number of nodes as the target network. The confidence score (bootstrap value) for an edge in the target network is estimated according to the chance of this edge appearing in networks under removal.

### Significance of the patient-survival gene network tested by a degree-approximating node randomisation

The significance of the patient-survival gene network is assessed by comparison to how often it would be expected by random. This comparison is done via a degree-approximating node/gene randomisation [21,22], which permutates gene labels but preserves node degrees. Similar to the robustness assessment above, for a permutated list of genes, a survival network is identified with the same/similar size as the target network. These networks identified via randomisation (100 times in this case) are used as a background to estimate the significance (*P* value) for each edge in the target network. These *P* values are also combined to a joint test for testing whether a global null hypothesis can be rejected, reporting an aggregated *P* value according to Fisher’s method (see the function ‘dPvalAggregate’ in the *dnet* package).

### Survival network-based landscape across tumour types

Relationships across tumour types are characterised based on genes in the patient-survival gene network and their mutation frequencies in each tumour type. *dnet* implements two methods for characterising sample relationships (here relationships across tumour types): one is to build a neighbour-joining tree, the other is to create a self-organising landscape. The first method takes as input a matrix of survival genes × tumour types, with each element for mutation frequency, and the built tree is attached with confidence values. The second method takes into account the connectivity in the network. The input matrix is about information on edges in the network, which are transformed from information on nodes: degree and mutation frequency. For an edge *e* and its two-end nodes *v*_*i*_ and *v*_*j*_, the information on the edge *e*^*k*^ for the tumour type *k* is transformed according to:

$$ {e}^k=\left|\frac{f_i^k}{d_i}\mathit{\hbox{-}}\frac{f_j^k}{d_j}\right| $$

where *d*_*i*_ and *d*_*j*_ are degrees for nodes *v*_*i*_ and *v*_*j*_, respectively, while ${f}_i^k$ and ${f}_j^k$ for their mutation frequencies in the tumour type *k*. Such a transformation considers mutation frequency absolute difference but being penalised by node degrees, thus fully utilising the information contained in the network. The transformed matrix of network edges × tumour types are used for self-organising tumour types onto a two-dimensional (2D) landscape (see the function ‘dNetReorder’ in the *dnet* package, which extends this functionality from package ‘supraHex’).

### Performance evaluation in identifying the patient-survival gene network

**Figure 2:**
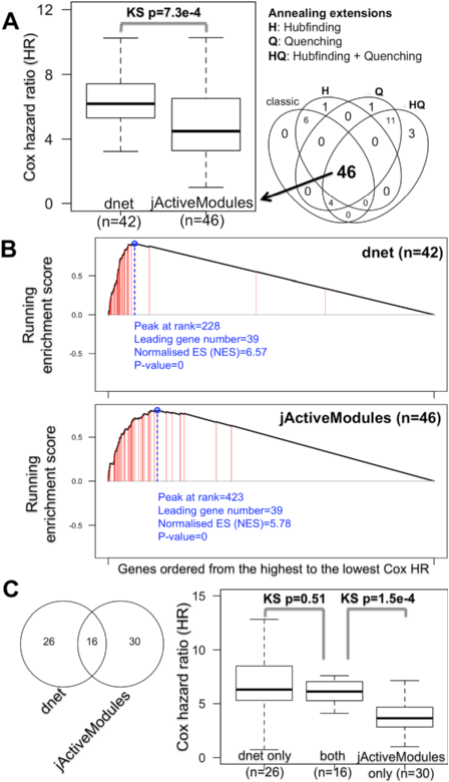
**Performance evaluation in identifying the patient-survival gene network.** The performance of the dent method is compared against a popular and commonly used method called ‘jActiveModules’ and its extensions. A two-sample Kolmogorov-Smirnov (KS) test is used to assess the significance (*P* value) of the differential distributions. **(A)** Boxplot displays the distribution of Cox hazard ratio (HR) for network genes. Also illustrated on the left are 46 genes in the consensus network identified by jActiveModules. This consensus is reached based on results from four different annealing options as indicated. **(B)** Gene set enrichment analysis (GSEA) of network genes. GSEA is used to examine the extent to which genes in a network are rank-enriched towards the highest Cox HR. The plots show the running enrichment score and a peak (circled in blue) with a normalised enrichment score (NES). Genes in the survival network are indicated with red lines. **(C)** Comparisons of survival genes identified by both methods. Left panel: Venn diagram illustrating the differences and intersections of survival genes. Right panel: boxplot illustrating the distribution of Cox HR grouped according to the Venn diagram.

The performance is evaluated against two state-of-the-art methods for identifying the patient-survival gene network using the same data input as before. The first is the method based on node scoring under beta-uniform mixture model [20,23], which is also implemented in our dnet package. Probably because this model does not fit the observed Cox *P* values, this method fails to identify any networks. The second method is based on simulated annealing, implemented as Cytoscape plug-in *jActiveModules* [24]. This method is commonly used [25] and thus suitable for performance comparisons. Owing to inability in controlling the node size, *jActiveModules* is sequentially applied to identify the smaller network from the previous larger one. Also owing to stochastic nature, *jActiveModules* is used with four different annealing options: the one is classic as reported in the original publication, and the other three using annealing extensions (hubfinding, quenching or both). Networks from these four annealing are merged into the consensus one consisting of nodes, comparable in size to the one identified by *dnet*. See Figure 2A for illustrations.

Patient-survival gene networks by *dnet* and *jActiveModules* are compared to each other in terms of performance on how well genes identified are survival-related. Performance on the survival relatedness is equivalent to how genes with the highest Cox HR (or the lowest Cox *P* values) tend to be in the identified network. Two performance measures are used to quantify this tendency. The first measure is the distribution (and the median) of Cox HR in the network. Difference in distributions is tested by two-sample Kolmogorov-Smirnov (KS) tests and measured by KS *P* value. The second alternative is the rank enrichment towards the highest Cox HR. It is measured by running enrichment score in gene set enrichment analysis (GSEA; also see the ‘Gene set enrichment analysis by *dGSEA*’ section). GSEA allows for visually and quantitatively determining the degree to which genes in the network are rank-enriched at the top of the whole genes list; this list is pre-ranked from the highest to the lowest Cox HR.

### Prognosic and druggable evaluation of the patient-survival gene network

Prognostic power is evaluated in two comparisons. First, distribution of Cox HR for each gene in the network is compared against the distribution for the same number of genes: (1) that are randomly chosen (naive baseline); (2) that are at the top list with the highest Cox HR (ideal baseline). Two-sample KS tests are used to assess the significance for their differential distributions. The second comparison is made for genes used individually or in combination, that is, HR for individual genes *versus* HR for genes in combination. Considering practical use, genes are combined in a way that an increasing number of genes are added in sequentially: genes with the more significant Cox *P* values always come first. Cox HR (and *P* value) for each gene combination is computed using age, gender and tumour type as covariates. The explanatory variable in subject is an aggregated mutation vector, telling how many mutations each patient has in terms of genes combined. Similarly, Cox HR distributions between individual genes and genes in sequential combination are evaluated by two-sample KS tests.

Druggable power is evaluated by enrichments of druggable gene categories (compiled from [26]). Hypergeometric distribution based enrichment analysis (also see the ‘Enrichment analysis by *dEnricher*’ Section) is applied to find druggable categories enriched in the network. Enrichment significance is defined under false discovery rate (FDR) of 0.05 or lower (after controlling multiple hypothesis tests).

### Gene set enrichment analysis by *dGSEA*

In the *dnet* package, the function ‘dGSEA’ implements a computational method for determining whether a gene set (for example, genes in the patient-survival gene network) appears randomly in a pre-ranked gene list or has a tendency to be at the top (or bottom) of this ranked list. Such tendency is quantified by running enrichment score, and in general by normalised enrichment score (NES) which is suitable for comparing results across different gene sets. The enrichment significance (*P* value) is assessed according to a null distribution that is estimated by randomly sampled gene sets (each sampled set has the same number of genes as the original set).

As described above, when determining the tendency of genes in the patient-survival network to have a higher Cox HR, the pre-ranked gene list is ordered according to Cox HR in a decreasing manner. In addition, *dGSEA* is also applied to look at the tendency of these survival genes to be at the top of the gene list pre-ranked according to: cross-tumour mutation ubiquity, mutation frequency within a single tumour type, and mutation numbers in each individual patient.

Our definition of cross-tumour mutation ubiquity is based on within-tumour-type mutation frequency. For a gene, mutation frequency within a tumour type is the proportion of patients having the mutated gene among all patients belonging to this tumour type. Mutation ubiquity defines how ubiquitous this mutation frequency is across tumour types. Let *f*_*i*_ be mutation frequency for a tumour type *i*, then mutation ubiquity *u* is formulated as:

$$ u=\left(\frac{{\displaystyle {\sum}_i^n{f}_i}}{\sqrt{{\displaystyle {\sum}_i^n{f}_i^2}}}\mathit{\hbox{-}}1\right)/\left(\sqrt{n}\mathit{\hbox{-}}1\right), $$

where *n* is the number of tumour types. This formula results in a mutation ubiquity ranging from 0 to 1. It approaches one when a gene is mutated in all tumour types with almost identical mutation frequency. At the other extreme, it takes a value of zero when a gene is only mutated in a single tumour type. Otherwise, mutation ubiquity interpolates smoothly between these two extremes.

### Enrichment analysis by *dEnricher*

The function ‘dEnricher’ implements enrichment analysis based on the hypergeometric distribution or Fisher’s exact test. It tests the significance of gene overlaps between a gene group (for example, genes in the patient-survival gene network) and gene annotations (for example, annotations by an ontology term, representing the known knowledge). The utility of *dEnricher* is to identify knowledge enrichments, thus giving a knowledge-relevance interpretation of this gene group. This relevance inference depends on which aspect of knowledge is used (see ‘Data in the *dnet* package’ section). It can evaluate the druggable relevance by using druggable gene categories as described above. When knowledge of gene evolutionary ages is provided, *dEnricher* can also be applied for analysis of evolutionary relevance. Briefly, it identifies our common ancestor in which a significantly higher number of survival genes first appeared. A more general utility lies in ontology enrichment analysis. Since terms in an ontology are structured as a hierarchy, *dEnricher* can also account for this hierarchal structure by respecting the parent-child dependency. Term enrichments are visualised in the context of the ontology hierarchy for better interpreting the relevance of the knowledge.

### Data in the *dnet* package

The backend of various analytical utilities supported in *dnet* is its built-in database spanning a wide range of the known gene-centric knowledge across well-studied organisms. Knowledge of genes can be: their interacting networks [19], annotations by various ontologies (in dcGO database [27,28]), evolutionary ages [29], and residual domain superfamilies [30]. They are provided as RData-formatted files, and are maintained and updated using in-house Perl scripts. For this study, we also populate the database by human-specific knowledge on druggable gene categories. All these are freely available at the package dedicated website [12].

## Results and discussion

### *dnet* uncovers an underlying patient-survival gene network across tumour types

**Figure 3:**
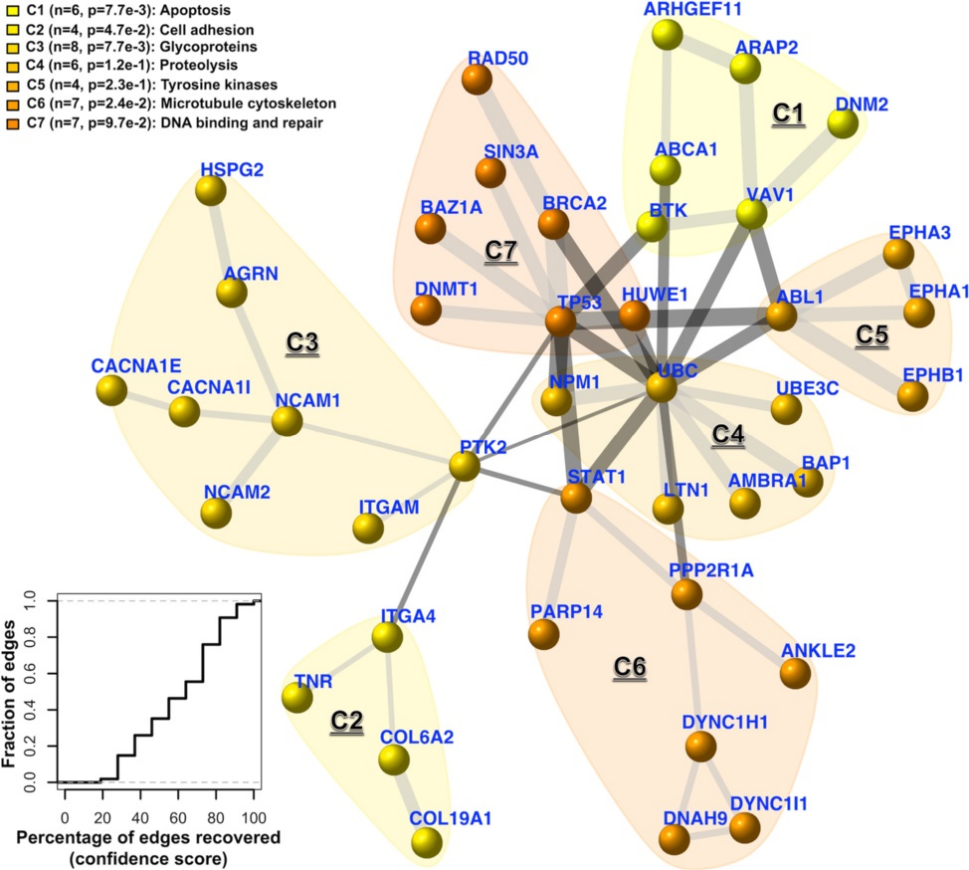
**A core patient-survival gene network, and its community structure and robustness.** This network contains 42 genes, most of which, upon mutated, are significantly correlated with patient survival (based on Cox proportional hazards model). The nodes/genes in the network are found to be naturally organised into seven communities (C1 to 7), each associated with distinct aspects of cancer biology. The significance (*P* value) of the communities is evaluated by two-sample Wilcoxon tests on two types of node degrees: within-community degrees *versus* between-community degrees. The thickness of an edge indicates the confidence score (under data removal). The bottom-left inset shows the cumulative distribution of the edge confidence scores.

In an attempt to discover gene networks controlling cancer patient survivalness, we developed an integrative method and implemented it as an open-source R package called ‘dnet’. By analysing the ‘TCGA’ mutation and clinical data of patients covering multiple tumour types [16], we uncovered a core gene network indicative of cross-tumour patient survival (Figure 3; also see Additional file 1). For ease of network content explanation and visualisation, we also analysed the community structure and identified seven communities (C1 to C7) inherent in the network. Although no prior knowledge was used for guiding community identification, interestingly we found these communities can be labelled with discernible biological processes, highly indicative of functional modular design. Notably, *TP53* in the DNA binding and repair community (C7) is directly linked to five communities including: the apoptosis community (C1), the tyrosine kinase community (C5), the proteolysis community (C4), the microtubule cytoskeleton community (C6), and the community containing genes all encoding glycoproteins (C3). *TP53* is indirectly linked to the cell adhesion community (C2), with *PTK2* in C3 as a connector. Community connectors like *PTK2* serve as a scaffold linking together communities, and thus are not necessarily correlated with the patient survival (see Additional file 2).

We tested the robustness of *dnet* in identifying the survival network. To do so, we systematically removed one tumour type at a time and measured how the survival network would respond to this data removal. For each edge in the network, we calculated a confidence score by estimating the likelihood of this edge getting recovered under removal (bootstrap value). As shown in the bottom-left corner of Figure 3, the edge confidence scores are relatively high, indicating that the recovery of edges is robust against input data removal and also the algorithm used. We see those edges falling within and connecting together survival-relevant communities (C7, C1, C5 and C4) are the most robust; they vary little upon data removal.

**Figure 4:**
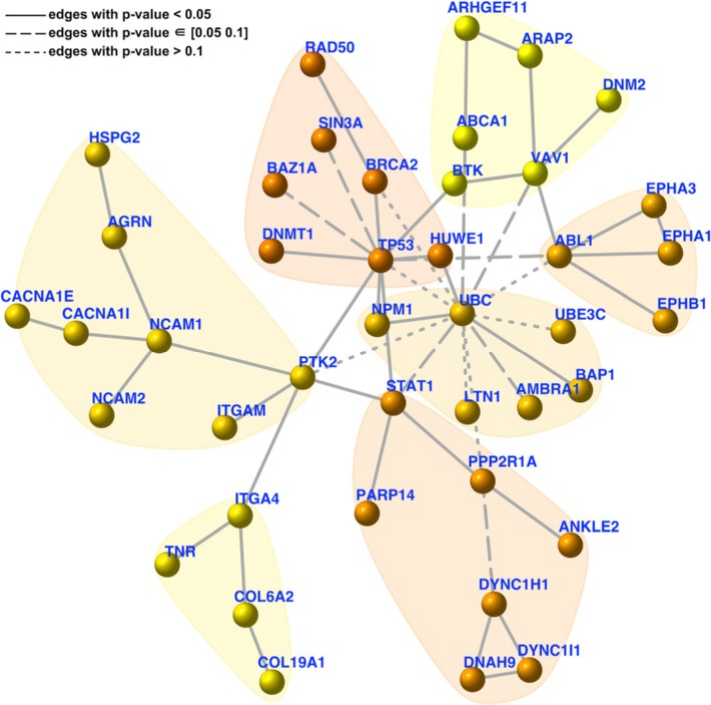
**Significance of the patient-survival gene network.** This network has the same network layout as in Figure 3, and the significance (*P* value; under data randomisation) of an edge is indicated by one of three different line styles.

We also tested the significance of the identified survival network using a novel data randomisation procedure. This randomisation shuttles gene/node labels but preserves node degrees in the network. For these randomisations, we identified survival networks (as background null distributions), from which we estimated how often (*P* value) each edge in the original network would be expected by random. The distribution of these edge *P* values is shown in Additional file 3; it can be seen that 78% of edges are true positives at a tolerable error rate of 0.05; this figure increases to 89% at an error rate of 0.1. This is an equivalent of an aggregated *P* value of 3.4e-28 when testing all edges as a whole according to Fisher’s method. Among those edges with high error rate (*P* value >0.1), most of them are between hubs, such as pairs of *UBC*-*PTK2*, *UBC*-*ABL1* and *UBC*-*TP53* (Figure 4). If these edges are removed, their terminal hubs still remain. There results strongly support two notions. First, the identified network is not a result of data bias but is of statistical significance. Second, such a degree-approximating randomisation excludes the possibility that hubs (for example, hotspot genes) would lead to a biased result. Admittedly, hubs tend to have edges between each other by chance, but their presence is required for connecting highly-isolated nodes (of interest).

**Figure 5:**
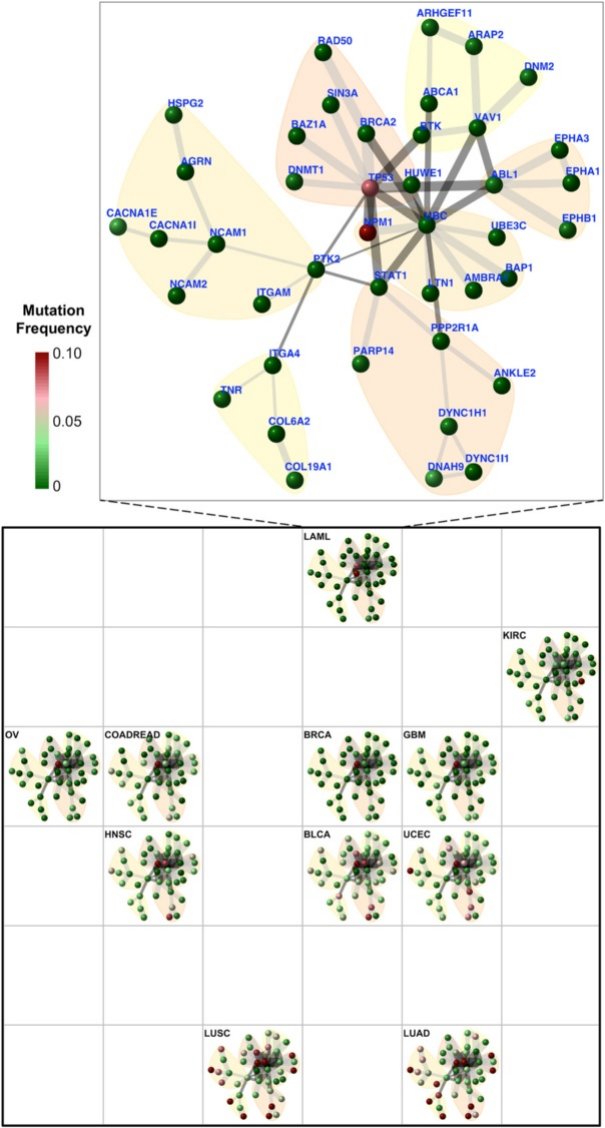
**The survival-network-based landscape of 11 tumour types.** Tumour types are self-organised onto a 6 × 6 grid based on mutation frequency and connectivity of genes/nodes in the survival network (see Methods for details). This grid represents the network-based 2D landscape, from which geometric locations delineate relationships between these 11 tumour types. Smaller squares illustrate the same network layout (as in Figure 3) but nodes are colour-coded according to tumour-type-specific mutation frequency information. The inset in the top shows the enlarged view for LAML. Abbreviations for these 11 tumour types are: bladder urothelial carcinoma (BLCA); breast adenocarcinoma (BRCA); colon and rectal carcinoma (COADREAD); glioblastoma multiforme (GBM); head and neck squamous cell carcinoma (HNSC); kidney renal clear cell carcinoma (KIRC); acute myeloid leukaemia (LAML); lung adenocarcinoma (LUAD); lung squamous cell carcinoma (LUSC); ovarian serous carcinoma (OV); uterine corpus endometrial carcinoma (UCEC).

To further understand this survival network, we looked at its ability to characterise relationships between tumour types. For this purpose, we developed a method fully exploiting information carried by the network (see Methods) and built a 2D landscape using a self-organising algorithm [14] (Figure 5). This landscape captures tumour type relationships, but also provides for displaying information (within-tumour-type gene mutation frequency on genes/nodes) to explain the observed relationships. For example, two tumour types of lung tissue of origins (LUAD and LUSC) locate at the bottom, with the highest proportion of survival genes frequently mutated. Another example is the leukaemia tumour type (LAML), in which genes *NPM1* and *TP53* are highly mutated. Because of this unique mutation profile, it is distinct in space from others. This simultaneous inspection of tumour type relationships and network information gives an intuitive clue as to what establishes the observed relationships. It should be noted, however, that any interpretation of the relationships are in the context of survivalness: mutation frequency and connectivity of genes in the survival network.

Survival advantage for patients within a tumour type can differ in age and gender, and these difference can be further complicated when comparing across tumour types. As illustrated in Additional file 4A, different tumour types have different survival curves: the poorest seen in GBM and LAML, and the most favourable in UCEC and BRCA. For these reasons, in survival analysis we incorporated three covariates (age, gender and tumour type) in our Cox regression model as the baseline (see Methods). With the survival network in hand, it is also interesting to examine the survivalness for each tumour type. Briefly, we used a Cox regression model taking age and gender as covariates in the baseline to compare against the full regression model. This full regression model includes an additional explanatory variable (a total number of mutations falling into the survival network), which is subjected to likelihood ratio tests (LRT). The Cox hazard ratio (HR) calculated from LRT is shown in Additional file 4B. From it, we see that this survival network is in general informative in characterising the survivalness observed in different tumour types. However, we also see that this cross-tumour survival network, though informative, is not simply so. For example, OV has a better survival curve than LUAD, but according to the network it has a higher HR (thus worse prognosis). It is reasonable since the identified survival network is not merely a reflection of the survivalness in individual tumour types, but also incorporates all possible combinatorial factors between age, gender and tumour types.

### *dnet* performs better in identifying the survival network than commonly used methods

We next evaluated the performance of the *dnet* method against a popular method implemented in Cytoscape plug-in *jActiveModules* [24] (although not originally designed for cross-tumour survival network identification). Comparisons were made using the same data input used by *dnet*. Performance results are only specific to this survival dataset since there is no gold-standard benchmark for evaluations. We found that *dnet* is superior over *jActivemodules* in identifying survival-related genes (Figure 2). First, *jActiveModules* requires a relatively complicated procedure (sequential running) for identifying the stable networks with desirable size. For this reason, we sought the best procedure in this application, and obtained the consensus network (see Methods). Second, genes identified by the *dnet* method tend to have higher Cox HR than those by the *jActiveModules* method (Figure 2A), and thus many more survival genes are identified by the *dnet* method. Indeed, survival genes are extremely enriched; this distinction is less extreme in the survival network identified by the *jActiveModules* method (Figure 2B). Looking further at genes commonly identified and unique to each method (Figure 2C; also see Additional file 5), we also see that genes unique to the *dnet* method have Cox HR at least comparable to genes commonly identified by both methods. In contrast, genes unique to the *jActiveModule* method tend to have lower Cox HR and are therefore of lesser survival-relatedness.

In the literature, a growing number of methods have been reported for network-based survival analysis [31–36]. Some of them have been implemented as tools, including *Net-Cox* [31], *Reactome FI* [32], *HyperModules* [36] and *HotNet* [35]. Among these, all but *Reactome FI* are conceptually similar to *dnet* in using survival data to guide survival network discovery, while *HyperModules* currently does not support the Cox regression required in this application. Unlike *dnet*, however, *Net-Cox* and *HotNet* both require sophisticated parameter tuning. A major limitation of these existing tools is that all of them are unable to adjust for covariables (such as age, gender and particularly tumour type); this is essential for cross-tumour survival analysis. Without adjustments for survival differences between different tumour types (as shown previously in Additional file 4A), these tools are only applicable in within-tumour-type survival network discovery. Therefore, here we do not provide direct comparisons. Instead, in Table 1 we provide a side-by-side comparative discussion in terms of availability, concept (supervised by survival data), covariable adjustments, analytical difficulty (parameter tuning) and other relevant aspects (visualisations, downstream interpretations and documentations). From the table, it becomes clear that *dnet* is most suitable for the task in cross-tumour survival network discovery and interpretations. This comparison also identifies a need for the adaptation of existing tools to fit this purpose.

**Table 1:** **Comparison of network-based survival analysis tools**

	***dnet***	***jActiveModules***	***Net-Cox***	***Reactome FI***	***HyperModules***	***HotNet***
**Platform/Language**	**R**	**Cytoscape**	**Matlab** ^**a**^	**Cytoscape**	**Cytoscape**	**Python**
**Operating Systems independent** ^**b**^	Yes	Yes	Yes	Yes	Yes	Linux/Unix
**Supervised by clinical survival data**	Yes	Yes	Yes	No	Yes^c^	Yes
**Able to adjust for covariables** ^**d**^	Yes^e^	Yes^e,f^	No	No	No	No
**Require sophisticated parameter setup**	No	Yes	Yes	No	No	Yes
**Advanced network visualisation**	Yes	Yes	No	Yes	Yes	Yes
**Downstream evolution analysis**	Yes	No	No	No	No	No
**With full documentations**	Yes	Yes	No	Yes	Yes	Yes

^a^Needs commercial license.

^b^Windows, Mac, Linux/Unix.

^c^Does not support Cox regression.

^d^Includes age, gender and tumour type that are required for cross-tumour survival analysis.

^e^It incorporates this functionality from the package ‘survival’.

^f^Can be extended for this purpose as shown in Figure 2.

### The survival network has potential for prognosis and druggability

**Figure 6:**
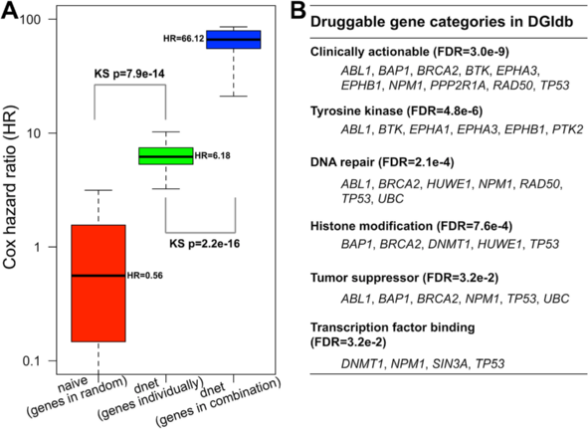
**The power of the patient-survival gene network in prognosis and druggability. (A)** Prognostic power. The boxplots display the distribution of Cox HR for: the randomly chosen 42 genes (as a naive method; in red), the 42 individual genes in the network (as identified by *dnet*; in green), and the genes in the network used in combination (in blue). Genes in the network used in combination yields an average of a 10-fold increase in Cox HR over using those genes individually (66.1 *versus* 6.2), which equates to a 100-fold gain in Cox HR using genes naively (66.1 *versus* 0.6). **(B)** Druggable power. The table lists significant enrichments of druggable gene categories and their annotated genes.

For the survival network to be of potential use, we next explored its power in prognosis and druggability. Prognostic power is seen when compared to the naive use (chosen at random); an average of 10-fold increases in Cox HR will be expected using genes in the survival network. If survival genes are combined for prognostic use, an average of 100-fold gain will be achieved (Figure 6A). It should be noted that genes in the survival network are not always on the top list of genes with the highest Cox HR (that is, an ideal list); this must be compromised to ensure that they are all interconnected cohesively as a whole, even though, we still see that these network genes, when used in combination, have a comparable prognostic power as seen in the combination of the ideal gene list (Additional file 6). These results collectively suggest the prognostic potential of this network signature.

For pharmaceutical use, it also requires evaluations of whether the survival genes are druggable. To address this power, we populated built-in data (in the *dnet* package) by druggable gene categories available from the Drug-Gene Interaction database [26], and performed enrichment analysis to examine the druggability of genes in the survival network. As shown in Figure 6B, the top enrichment is a category ‘Clinically actionable’, and other enrichments cover a wide spectrum of anti-neoplastic categories. Clinically actionable genes include: genes encoding tyrosine kinases (*ABL1*, *BTK*, *EPHA3*, *EPHB1*), genes involved in DNA repair (*ABL1*, *BRCA2*, *NPM1*, *RAD50*, *TP53*) and histone modification (*BAP1*, *BRCA2*, *TP53*), and tumour suppressor genes (*ABL1*, *BAP1*, *BRCA2*, *NPM1*, *TP53*). Taken together, genes in the survival network have the potential of being used in combination for better prognosis, and of being targeted for clinical use.

In addition to the cross-tumour survival network, we also applied *dnet* to identify cancer-specific survival networks and to investigate their characteristics for druggability. As expected, these cancer-specific networks differ from each other in the genes involved and in node size, with the lowest (5) in LAML to the highest (60) in LUSC (see Additional file 7 for details). This is consistent with the mutated gene number observed in each of tumour types. When looking at the druggability of genes in each cancer-specific network, unexpectedly we found that they are all clinically actionable (Additional file 8). We also found that most of cancer-specific networks share similar druggable gene category enrichments as observed in the core cross-tumour survival network. These results suggest that different tumour types vary greatly in survival-related genes; however, they probably utilise several common characteristics to constrain these survival genes. This also indicates the feasibility of deriving commonality from varied tumour types.

### The survival network offers an overview of inter-tumour mutation similarity and intra-tumour mutation heterogeneity

**Figure 7:**
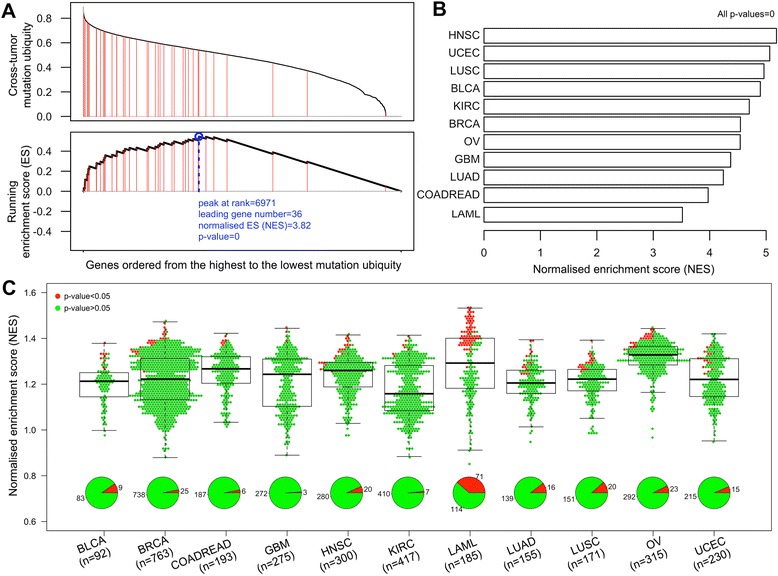
**A survival network based overview of inter-tumour mutation similarity and intra-tumour mutation heterogeneity.** Gene set enrichment analysis (GSEA) is applied to the genes in the survival network for: cross-tumour mutation ubiquity **(A)**, mutation frequency within a single tumour type **(B)**, and mutation numbers in each individual patient **(C)**. The top graph of **(A)** shows mutation ubiquity values (y-axis) for genes ranked from the highest to the lowest values (x-axis). Genes in the survival network are indicated by red lines. The graph below shows the running enrichment score and a peak (circled in blue) with a normalised enrichment score (NES) and *P* value. GSEA is also applied to analyse each of the 11 tumour types **(B)** and each of 3,096 individual patients **(C)**. Each dot in **(C)** stands for an individual patient, with red for significant enrichment and green otherwise. Abbreviations for tumour types are the same as described in Figure 5.

Viewing genes in the survival network as a signature, we conducted gene set enrichment analysis (GSEA) to look at the extent to which these genes are enriched in terms of cross-tumour mutation ubiquity, mutation frequency within a single tumour type, and mutation numbers for each individual patient (Figure 7). Our definition of cross-tumour mutation ubiquity is based on within-tumour-type mutation frequency, defined as how ubiquitous this mutation frequency is observed across tumour types. Figure 7A shows the rank distribution of survival genes in terms of mutation ubiquity, displaying a tendency towards higher mutation ubiquity. We also see such a tendency for all tumour types in terms of within-tumour-type mutation frequency (Figure 7B). These results suggest that there is a high degree of inter-tumour similarity in the relevance to survival. In sharp contrast, GSEA analysis of 3,096 individual patients reveals a high degree of intra-tumour heterogeneity for their relatedness to the survival network (Figure 7C). These results add a new layer of information: survivalness, to explain our current observations of inter-tumour mutation similarity and intra-tumour mutation heterogeneity [37,38].

### The survival network is traceable in evolution, most prominently to our ancestor Deuterostomia

**Figure 8:**
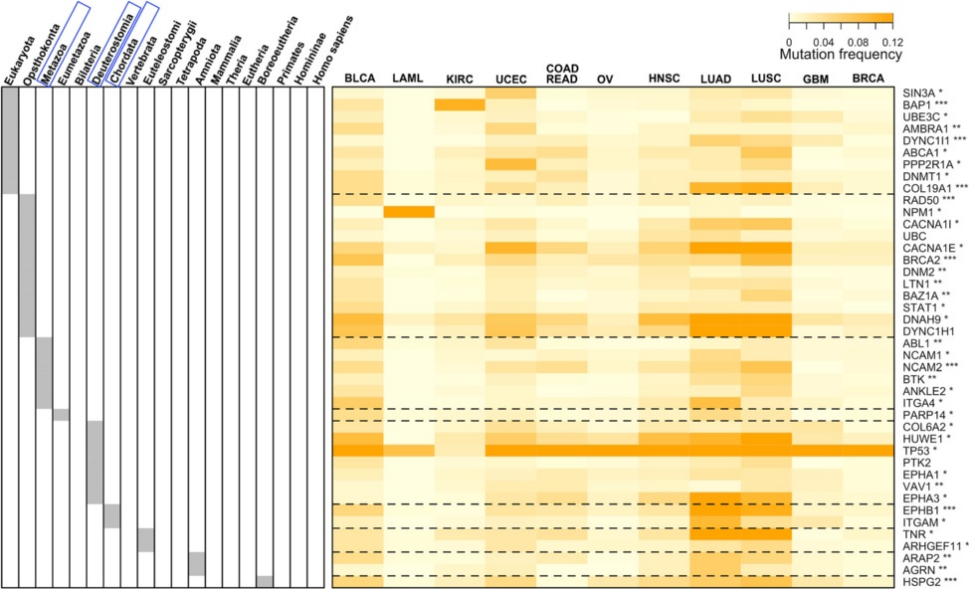
**Evolutionary age of genes in the survival network.** This is a heatmap of the mutation frequency of genes (in rows) *versus* tumour types (in columns). The tumour types are ordered based on a neighbour-joining tree (built from the mutation frequency matrix). The evolutionary history of genes is based on species tree of life (sTOL [29]). The left panel indicates the ancestor in which genes first appeared. Ancestor enrichment analysis shows that these survival genes contain a significantly higher number of genes first created at Metazoa, Deuterostomia and Chordata (framed in blue; FDR <0.05). Indicated beside the gene symbols are their Cox *P* values: *<0.05; **<0.01 and ***<0.005. Abbreviations for tumour types are the same as described in Figure 5.

Since the survival network controls cancer patient survivalness, we therefore further explored the possibility of tracing its evolutionary origins. For such a purpose, we studied the evolutionary history of survival genes using our previously built species tree of life (sTOL) [39]. This tree spans all completely sequenced genomes and best consolidates the knowledge of structural genomics and the NCBI taxonomy [29]. From sTOL, we retrieved gene evolutionary/phylostratific age information based on the first creation. For a human gene, evolutionary age is determined by the ancestor in which this gene was first created. Figure 8 illustrates the survival genes and their corresponding first-created ancestors. We see that half of survival genes were created at the animal-fungi boundary (Opisthokonta) or earlier; this proportion increases to 65% at or before the ancestor of animals (Metazoa), and up to 83% at our ancestors as early as Deuterostomia. To test whether when survival genes were created is a random event or has a preference, we conducted enrichment analysis using gene repertoires at each ancestor as background. Ancestor enrichments show that survival genes did not appear randomly in their evolutionary history but were preferentially created at these ancestors: Metazoa, Deuterostomia and Chordata (FDR < 0.05; see also Additional file 9).

**Figure 9:**
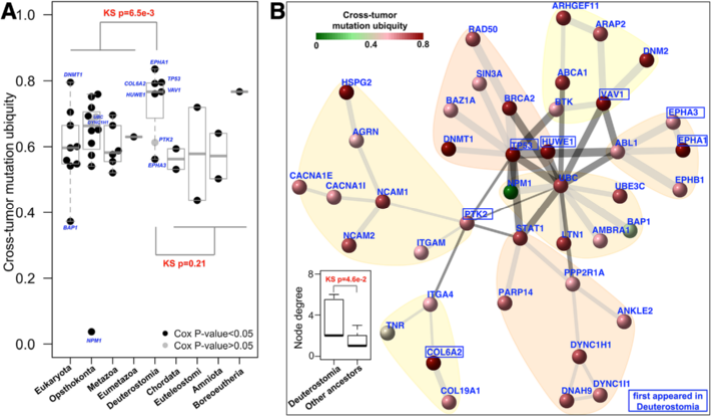
**Genes first appearing in Deuterostomia mutate more ubiquitously and tend to be hubs. (A)** Boxplot of genes in the survival network. Dots represent genes, color-coded according to their Cox *P* values. These genes are grouped according to ancestor in which they first appeared (x-axis). Cross-tumour mutation ubiquity (y-axis) measures how ubiquitous the mutation rate of a gene is in different tumour types. **(B)** Visualisation of the survival network with nodes/genes color-coded by mutation ubiquity. Also indicated are genes first appeared in Deuterostomia. The bottom-left inset shows the differential distributions of node degrees for genes first appeared in Deuterostomia against in our other ancestors.

When simultaneously displaying the mutation frequency matrix and their first-created ancestors for survival genes (Figure 8), we notice a tendency for Deuterostomia-originated genes to mutate ubiquitously across tumour types. To quantify such a tendency, we introduced the concept of cross-tumour mutation ubiquity. Based on it, we compared the distribution for survival genes grouped according to their first appearance in ancestors (Figure 9A). We see that there is a clear difference in mutation ubiquity between genes first appearing in Deuterostomia and genes created earlier (KS tests; *P* = 6.5e-3), but no significant support for the difference to genes appearing thereafter (*P* = 0.21). Figure 9B provides an integrated view of relationships between mutation ubiquity, ancestors and connectivity (degree) of survival genes. From it, we also see a significant difference in node connectivity between Deuterostomia and other ancestors.

Evolutionary tracking analysis of cancer genes has previously suggested the importance of Metazoa in the emergence of oncogenes and tumour suppressor genes [40]. Conceptually, this is consistent with our observations: many cancer-survival genes first appeared in Metazoa. Beyond this, our results also point to the evolutionary significance of a more recent ancestor Deuterostomia in shaping up genes related to cancer patient survivalness. Our work adds a new layer of information for cancer evolutionary analysis: survival-relatedness. Cancer survival genes continued to emerge after Metazoa, and probably not coincidentally at Deuterostomia. Deuterostomia are characterised by their plastic ability in cell-fate determination that they acquired but is absent in preceding ancestors [41]. The most parsimonious explanation for acquisition of such plasticity is to create *de novo* highly mutable genes with broad effects on cell fate. Partially because of the high mutability in different cell/tumour types, and in part because of their essentiality in evolution, we postulate that genes first created at Deuterostomia are more likely to be now affecting cancer patient survivalness. Some of these genes have been highly studied, such as *TP53* and its degrader *HUWE1*, and protein-tyrosine kinase signals such as *EPHA1* and *EPHA3.* Others are less studied, such as *PTK2* and *VAV1*, and experimental data have suggested so [42]. Our evolutionary analysis strongly suggests that they are more important in the study of cancer than currently appreciated.

## Conclusions

Applying *dnet* in analysing all of the ‘TCGA’ mutation and clinical data of >3,000 patients covering multiple tumour types (Figure 1), we uncovered a network of genes (Figure 3) for which most of their mutations are significantly correlated with patient survival. This survival network has community structure responsible for distinct aspects of tumour hallmarks. It is insensitive to the removal of single tumour types (Figure 3), is not an artifact of data characteristics (as shown by randomisation in Figure 4), can be used for characterising relationships between tumour types (Figure 5) and is generally informative for characterising survivalness for individual tumour types as well. The kind of survival network would not be identified via other commonly used methods (Figure 2) or indeed attempted (Table 1). Genes in this core network seem to be far more informative for prognosis when used in combination than when used individually, and are potentially druggable in the clinic (Figure 6); we also show this druggability is universal for cancer-specific survival networks even though their gene members differ greatly. Using these genes as a signature, we examined the distribution of mutations at three different levels of granularity (across tumour types, within a single tumour type, and in individual patients); Figure 7 shows the relevance to survival of intra-tumour heterogeneity and inter-tumour similarity. Strikingly, we also observe a clear relationship between cross-tumour mutation frequency and the evolutionary age of these survival genes (Figure 8). We observe a significantly higher mutational heterogeneity across cancer types in genes first created in Deuterostomia (Figure 9). Consistent with plastic cell-fate determination first appearing in this ancestor, we postulate that highly mutable genes with broad effects on cell fate were created at this time. Some have been highly studied such as *TP53* and its degrader *HUWE1*, and genes involved in protein-tyrosine kinase signals such as *EPHA1* and *EPHA3.* We also identify the less-studied *PTK2* and *VAV1* genes in the same category, and suggest that they are more important in the study of cancer survivalness than currently appreciated.

Our findings support the hypothesis that ‘survivalness’ is at least partly under the control of networks of mutated genes that also are traceable in evolution. This survival network has the potential to directly influence clinical practice in developing better prognostic, diagnostic and therapeutic protocols. The open source R package we present here is available for anybody to reproduce this work or apply our methodology to analyse other emerging mutation and clinical data on cancer patient survival.

## Additional files

Additional file 1:
**Genes in the patient-survival gene network and their relevant information.**
Additional file 2:
**The patient-survival gene network with nodes color-coded according to Cox hazard ratio (A) and Cox** ***P*** **value (B).**
Additional file 3:
**Distribution of** ***P*** **values for edges in the patient-survival gene network.**
Additional file 4:
**Survivalness for individual tumour types.** **(A)** Kaplan-Meier survival curves. Notably, the survival curve for each tumour type does not adjust for age and gender, and these curves are used to illustrate differentiated survival advantages for different tumour types. **(B)** Bar plot of Cox hazard ratio (HR) for different tumour types according to the survival network. For each tumour type, the calculation of HR has already adjusted for age and gender (as covariates; within the baseline regression), and is to test for an additional explanatory variable: a total number of mutations falling into the survival network. Additional file 5:
**Comparing the survival genes identified by** ***dnet*** **and** ***jActiveModules*** **.**
Additional file 6:
**Comparing the prognostic power of 42 network genes identified by** ***dnet*** **and 42 top genes with the highest Cox HR (that is, an ideal gene list as baseline).** The boxplots display the distribution of Cox HR for these genes used individually or in combination. As expected, network genes identified by *dnet* are not always on the top gene list with the highest Cox HR (6.2 *versus* 9.5, with their ratio: 0.65). This is necessary to make sure that all these network genes are interconnected; it differs from the ideal baseline in which genes have highest Cox HR but lack cohesiveness as a whole. When used in combination, however, network genes still have a comparable prognostic power as the ideal gene list (66.1 *versus* 79.7, with their ratio: 0.83). Additional file 7:
**Cancer-specific survival networks.** This file contains 11 sheets, each for a tumour type in which member genes and their Cox information are listed and visualised. Additional file 8:
**Enrichments of druggable gene categories in cancer-specific survival networks.**
Additional file 9:
**Ancestor enrichments of genes in the patient-survival gene network.**

## Abbreviations

BLCA: Bladder urothelial carcinoma
BRCA: Breast adenocarcinoma
COADREAD: Colon and rectal carcinoma
FDR: False discovery rate
GBM: Glioblastoma multiforme
GSEA: Gene set enrichment analysis
HNSC: Head and neck squamous cell carcinoma
HR: Hazard ratio
KIRC: Kidney renal clear cell carcinoma
KS: Kolmogorov-Smirnov
LAML: Acute myeloid leukaemia
LRT: Likelihood ratio tests
LUAD: Lung adenocarcinoma
LUSC: Lung squamous cell carcinoma
OV: Ovarian serous carcinoma
sTOL: Species tree of life
UCEC: Uterine corpus endometrial carcinoma
