# Supplementary figures and images for: The ‘dnet’ approach promotes emerging research on cancer patient survival
Source: Genome Med. 2014 Aug 26;6(8):64. doi: 10.1186/s13073-014-0064-8 (PMC4160547; doi:10.1186/s13073-014-0064-8)

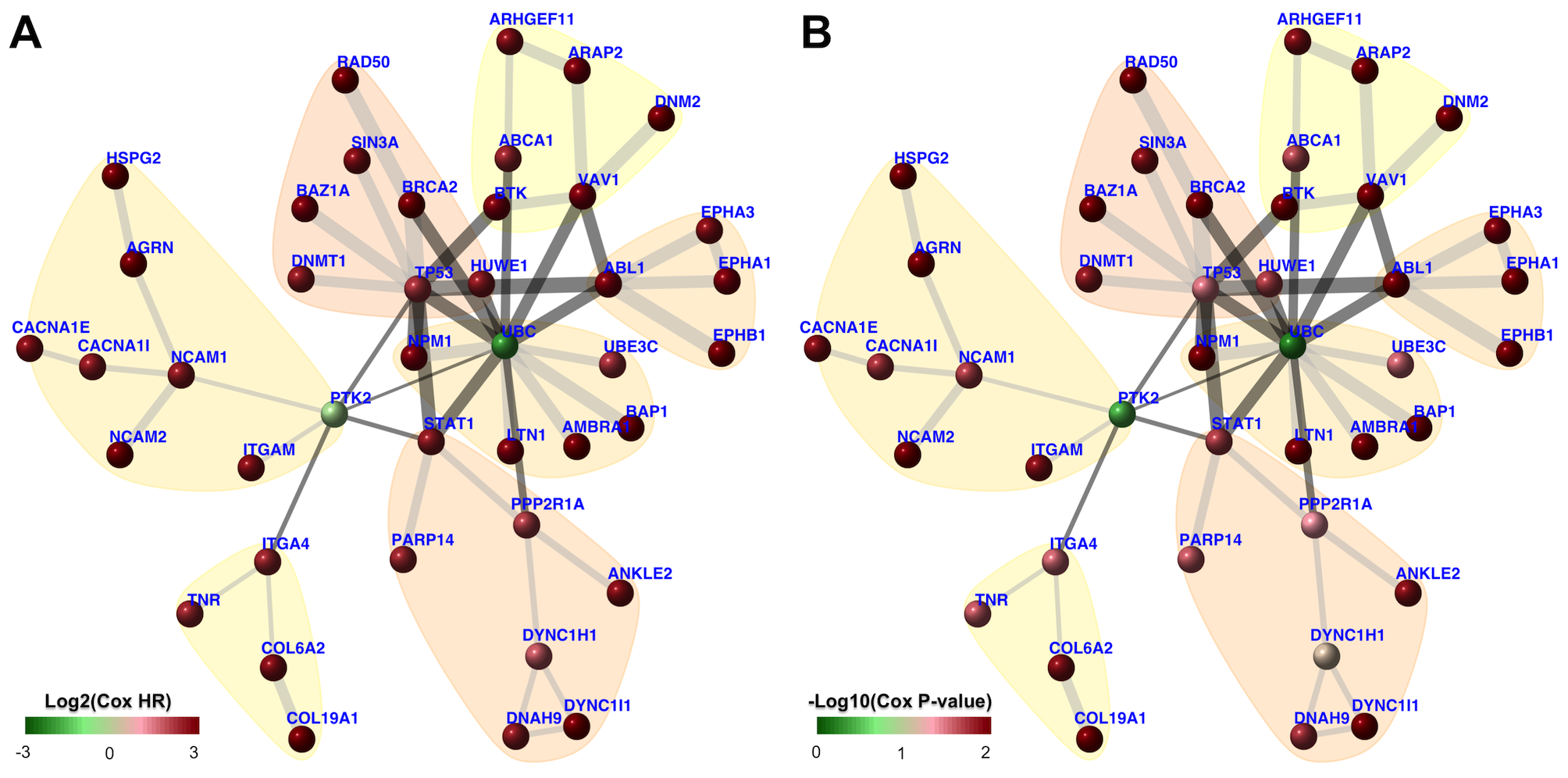

Supplement: Additional file 2: — The patient-survival gene network with nodes color-coded according to Cox hazard ratio (A) and Cox P value (B). [file 13073_2014_64_MOESM2_ESM.png]

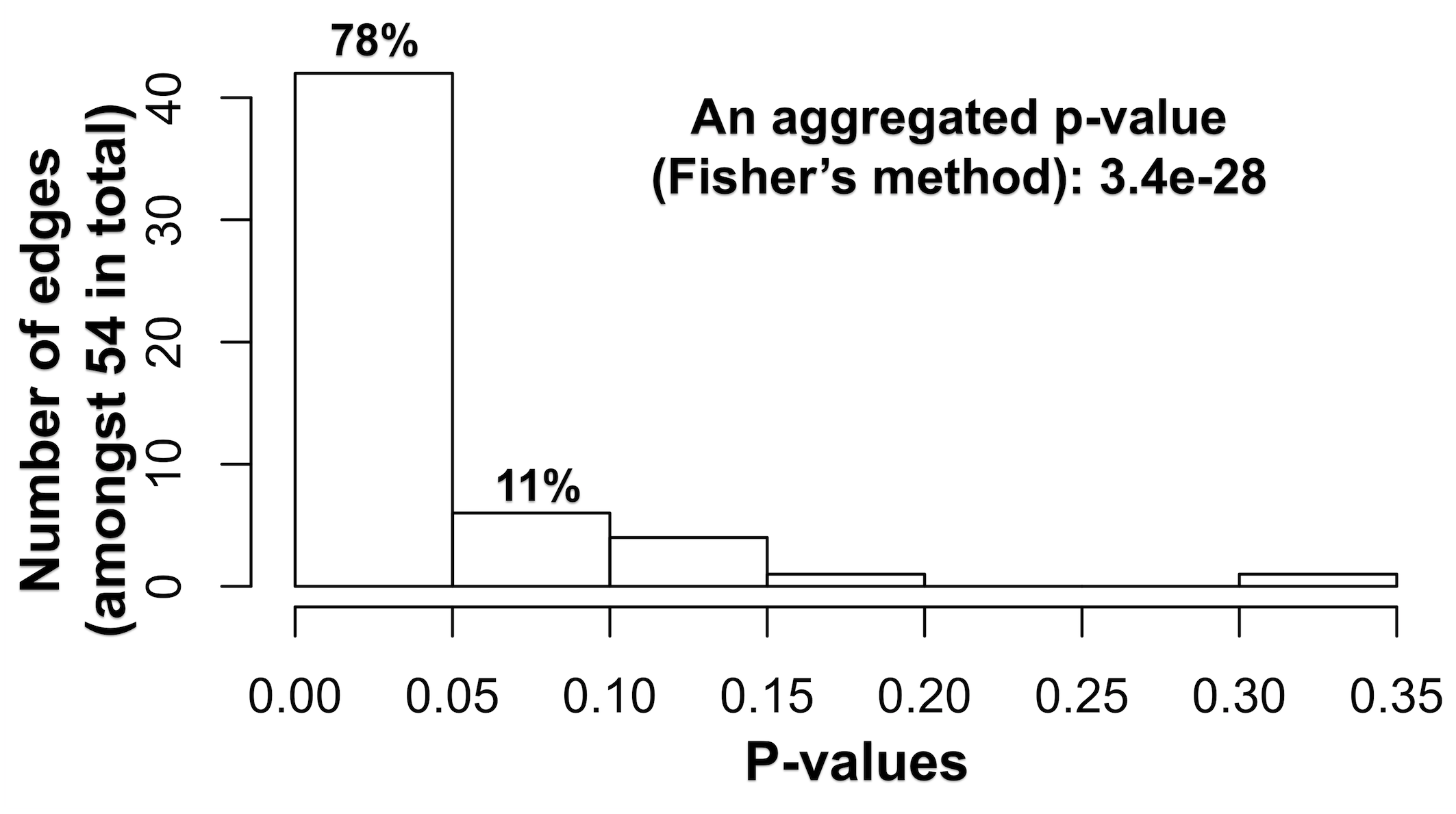

Supplement: Additional file 3: — Distribution of P values for edges in the patient-survival gene network. [file 13073_2014_64_MOESM3_ESM.png]

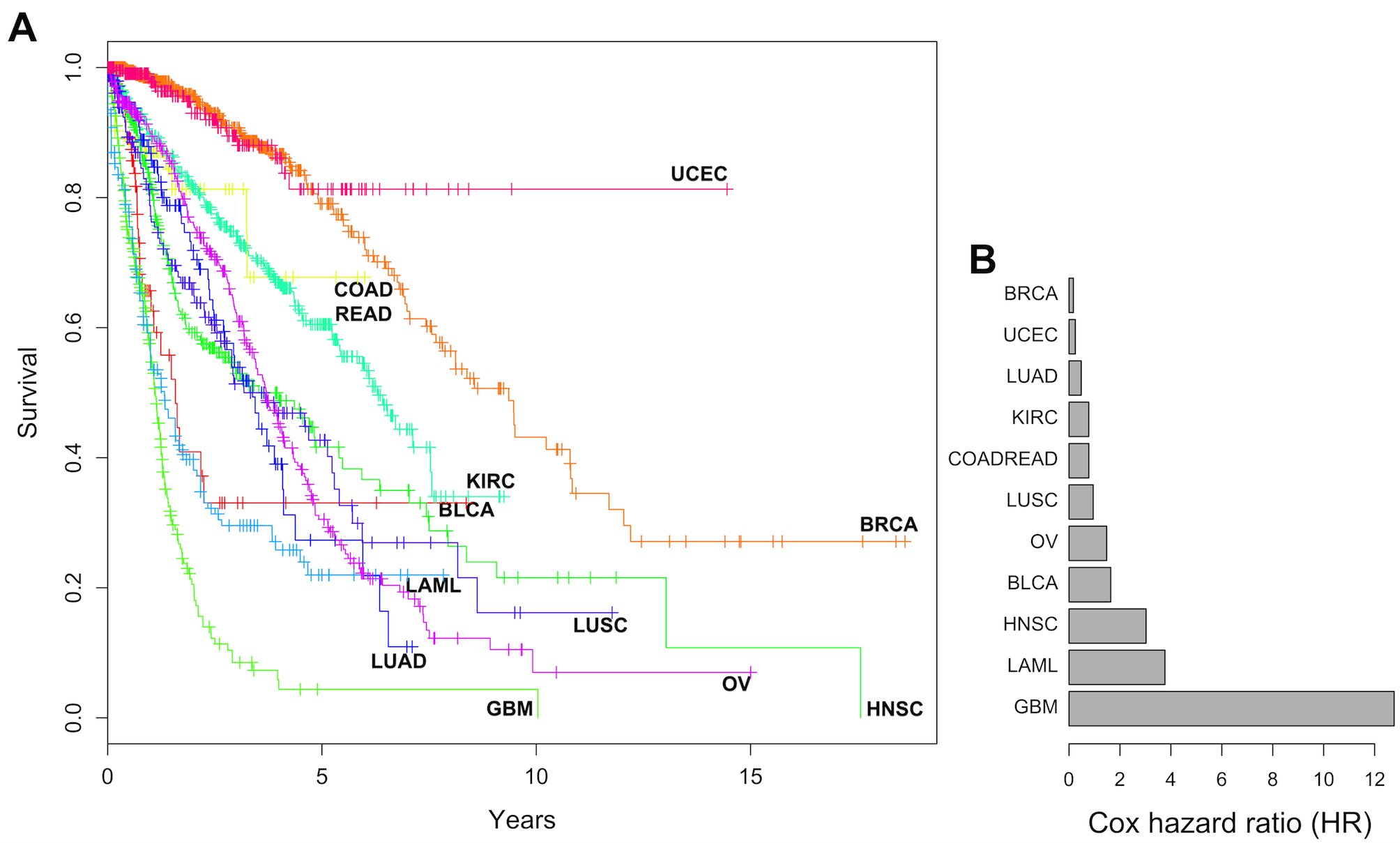

Supplement: Additional file 4: — Survivalness for individual tumour types. (A) Kaplan-Meier survival curves. Notably, the survival curve for each tumour type does not adjust for age and gender, and these curves are used to illustrate differentiated survival advantages for different tumour types. (B) Bar plot of Cox hazard ratio (HR) for different tumour types according to the survival network. For each tumour type, the calculation of HR has already adjusted for age and gender (as covariates; within the baseline regression), and is to test for an additional explanatory variable: a total number of mutations falling into the survival network. [file 13073_2014_64_MOESM4_ESM.png]

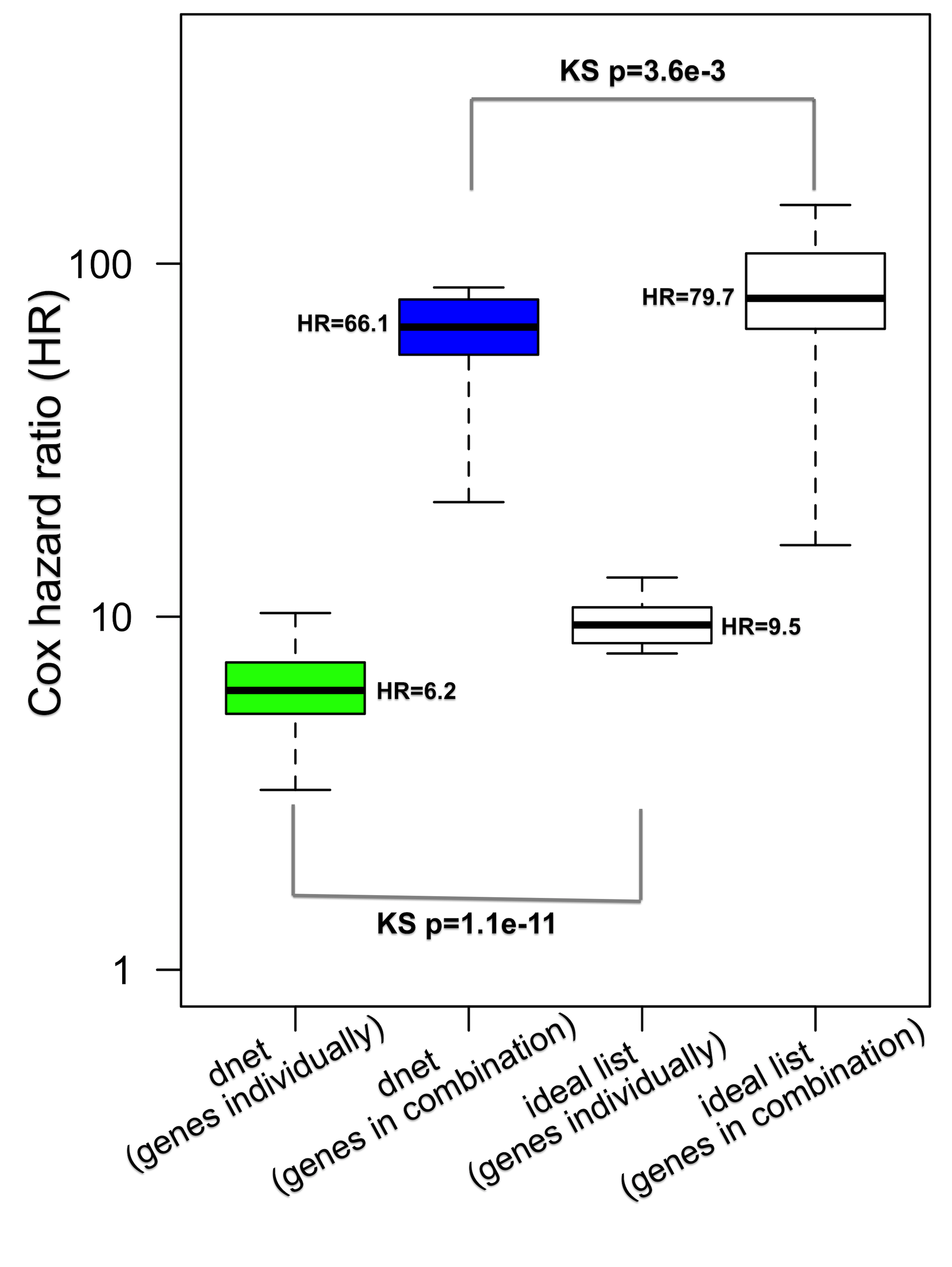

Supplement: Additional file 6: — Comparing the prognostic power of 42 network genes identified by dnet and 42 top genes with the highest Cox HR (that is, an ideal gene list as baseline). The boxplots display the distribution of Cox HR for these genes used individually or in combination. As expected, network genes identified by dnet are not always on the top gene list with the highest Cox HR (6.2 versus 9.5, with their ratio: 0.65). This is necessary to make sure that all these network genes are interconnected; it differs from the ideal baseline in which genes have highest Cox HR but lack cohesiveness as a whole. When used in combination, however, network genes still have a comparable prognostic power as the ideal gene list (66.1 versus 79.7, with their ratio: 0.83). [file 13073_2014_64_MOESM6_ESM.png]
