# Supplementary material for: The ‘dnet’ approach promotes emerging research on cancer patient survival
Source: Genome Med. 2014 Aug 26;6(8):64. doi: 10.1186/s13073-014-0064-8 (PMC4160547; doi:10.1186/s13073-014-0064-8)
Supplement: Additional file 8: — Enrichments of druggable gene categories in cancer-specific survival networks. [file 13073_2014_64_MOESM8_ESM.pdf]

Druggable gene categories in DGIdb	Enrichment significance		
	Z-score	P-value	FDR
BLCA-specific survival network			
Clinically actionable	4.24	2.8E-04	2.2E-03
Tyrosine kinase	4.49	2.6E-04	2.2E-03
Transcription factor binding	3.57	8.1E-04	4.3E-03
Ion channel	2.78	4.2E-03	1.5E-02
Transcription factor complex	2.77	4.6E-03	1.5E-02
Histone modification	2.05	1.6E-02	4.3E-02
BRCA-specific survival network			
Clinically actionable	3.47	1.5E-03	1.0E-02
Tumor suppressor	2.86	3.0E-03	1.1E-02
DNA repair	2.38	8.7E-03	2.0E-02
COADREAD-specific survival network			
Clinically actionable	4.88	1.2E-04	1.5E-03
Histone modification	3.35	1.8E-03	1.2E-02
Tyrosine kinase	2.95	3.9E-03	1.7E-02
DNA repair	2.38	8.7E-03	2.2E-02
External side of plasma membrane	2.45	7.9E-03	2.2E-02
Transcription factor binding	2.29	1.0E-02	2.2E-02
Transcription factor complex	1.78	2.2E-02	4.2E-02
GBM-specific survival network			
Clinically actionable	6.25	4.6E-06	6.0E-05
Tyrosine kinase	3.79	1.0E-03	6.5E-03
Ion channel	2.55	6.5E-03	2.8E-02
HNSC-specific survival network			
Clinically actionable	7.38	1.9E-07	2.5E-06
DNA repair	4.53	9.1E-05	5.9E-04
Tyrosine kinase	4.49	2.6E-04	1.1E-03
Kinase	3.41	7.4E-04	2.4E-03
Nuclear hormone receptor	3.86	1.2E-03	3.2E-03
Transcription factor binding	2.76	4.4E-03	9.6E-03
Drug resistance	2.27	1.1E-02	2.1E-02
Transcription factor complex	1.81	2.4E-02	3.9E-02
KIRC-specific survival network			
Clinically actionable	6.28	6.8E-06	3.7E-05
Tumor suppressor	5.37	4.6E-06	3.7E-05
Histone modification	4.72	1.5E-04	5.4E-04
Tyrosine kinase	4.71	2.5E-04	6.9E-04
Transcription factor binding	3.38	1.4E-03	3.0E-03
DNA repair	2.38	8.7E-03	1.6E-02
Transcription factor complex	1.78	2.2E-02	3.5E-02
Kinase	1.75	2.7E-02	3.7E-02

LAML-specific survival network			
Clinically actionable	10.50	0.0E+00	0.0E+00
Tumor suppressor	5.80	0.0E+00	0.0E+00
DNA repair	5.97	1.1E-05	2.5E-05
Tyrosine kinase	6.53	4.1E-05	7.1E-05
Transcription factor binding	3.72	8.1E-04	1.1E-03
Kinase	2.34	6.3E-03	7.4E-03
LUAD-specific survival network			
Clinically actionable	7.02	6.4E-07	7.7E-06
Drug resistance	6.42	1.0E-06	7.7E-06
Kinase	3.36	8.6E-04	4.3E-03
Histone modification	2.38	9.2E-03	3.0E-02
Transcription factor binding	2.31	1.0E-02	3.0E-02
Transcription factor complex	2.13	1.4E-02	3.0E-02
Tyrosine kinase	2.13	1.3E-02	3.0E-02
LUSC-specific survival network			
Clinically actionable	8.07	1.6E-08	2.7E-07
DNA repair	5.83	2.2E-06	1.9E-05
Phosphatidylinositol 3 kinase	8.18	6.0E-06	3.4E-05
Tumor suppressor	4.55	3.2E-05	1.3E-04
Kinase	3.54	5.0E-04	1.7E-03
Serine threonine kinase	2.44	8.1E-03	2.3E-02
OV-specific survival network			
Clinically actionable	7.73	5.5E-08	6.1E-07
Histone modification	5.59	1.2E-05	6.4E-05
Transcription factor binding	4.67	5.5E-05	2.0E-04
Cell surface	2.73	4.5E-03	9.9E-03
Tyrosine kinase	2.85	4.4E-03	9.9E-03
Tumor suppressor	2.16	1.3E-02	2.5E-02
DNA repair	1.73	3.0E-02	4.7E-02
UCEC-specific survival network			
Clinically actionable	6.03	1.1E-05	1.3E-04
Nuclear hormone receptor	5.26	2.5E-04	1.5E-03
External side of plasma membrane	3.84	8.7E-04	3.5E-03
Cell surface	2.85	3.6E-03	1.1E-02
Drug resistance	2.45	7.8E-03	1.9E-02
